# Dual-Branch Discrimination Network Using Multiple Sparse Priors for Image Deblurring

**DOI:** 10.3390/s22166216

**Published:** 2022-08-18

**Authors:** Jialuo Li, Shichao Cheng, Yueqiang Tao, Huasheng Liu, Junzhe Zhou, Jianhai Zhang

**Affiliations:** 1 School of Computer Science and Technology, Hangzhou Dianzi University, Hangzhou 310018, China; 2 Key Laboratory of Brain Machine Collaborative Intelligence of Zhejiang Province, Hangzhou 310018, China; 3 Shangyu Institute of Science and Engineering, Hangzhou Dianzi University, Shaoxing 310005, China

**Keywords:** image deblurring, multiple sparse priors, dual-branch GAN, image restoration

## Abstract

Blind image deblurring is a challenging problem in computer vision, aiming to restore the sharp image from blurred observation. Due to the incompatibility between the complex unknown degradation and the simple synthetic model, directly training a deep convolutional neural network (CNN) usually cannot sufficiently handle real-world blurry images. An existed generative adversarial network (GAN) can generate more detailed and realistic images, but the game between generator and discriminator is unbalancing, which leads to the training parameters not being able to converge to the ideal Nash equilibrium points. In this paper, we propose a GAN with a dual-branch discriminator using multiple sparse priors for image deblurring (DBSGAN) to overcome this limitation. By adding the multiple sparse priors into the other branch of the discriminator, the task of the discriminator is more complex. It can balance the game between the generator and the discriminator. Extensive experimental results on both synthetic and real-world blurry image datasets demonstrate the superior performance of our method over the state of the art in terms of quantitative metrics and visual quality. Especially for the GOPRO dataset, the averaged PSNR improves 1.7% over others.

## 1. Introduction

Image deblurring is one of the crucial problems in the computer vision and image processing community. The blurry image is caused by camera shake or object movement while taking a long time exposure photo. It causes problems when dealing with high-level tasks such as object detection and tracking [1,2,3,4,5,6]. Image deblurring is a task restoring a sharp image from a given blurred observation.

In traditional deblurring research, the blurry image is simulated as a blur kernel related to motion trajectory acting on a potentially clear sharp image as follows,

(1) b=k⊗x+n

where k, x and b denote blur kernel, sharp image and blurred image, respectively; n is the added Gauss noise, and ⊗ denotes the convolution operation. It is a highly ill-posed problem since both kernel and sharp image are unknown. To restore a latent sharp image, most of the works tend to build an optimization model (Equation (2)) with a variety of image priors as constraining terms based on the maximum a posteriori (MAP) framework.

(2) $$\arg\min_{k,x}{∥k⊗x-b∥}_{2}^{2}+p\left(x\right)+p\left(k\right)$$

where p(x) and p(k) are the constraints of latent sharp image and blur kernel, respectively. Total variation [7], hyper-Laplacian [8], dark channel [9] and other image priors are proposed as p(x) to describe the distribution characteristics of the sharp image different from the blurry image. By solving the optimal model in Equation (2), these methods have better performance on synthetic datasets, including the blurry image generated by the hypothesis in Equation (1). However, for the real blurry image with complex degradation, it is hard to establish a corresponding complex optimization model and solve it directly, which leads to the unsatisfied restoration of the real-world blurry image.

To simulate the complex degradation, some works regard the blurred image as the integration of a series of successive multi-frame instant and sharp snapshots during the exposure duration from a high-speed camera. By discrete integration, Nah et al. [10] proposed a new large-scale dataset including pairs of blurry images and the corresponding sharp image. Based on this dataset, numerous deep-learning-based methods for image deblurring have been proposed by training various network architectures to simulate the complex imaging inverse processing of blurry images [10,11,12,13]. Nah et al. [10] proposed a multi-scale convolutional neural network that restores sharp images in an end-to-end manner. Following the multi-scale strategy, Tao et al. [11] presented a scale-recurrent network for the deblurring task and produced better quality results. These deep approaches perform better than traditional methods on the discretized synthetic blurred image. Unfortunately, these deep approaches rely on training data distribution so that the gap between the real-world blurry image and the discretized synthetic blurry image cannot decline.

To alleviate the dependence on training data while simulating complex degradation, GAN-based methods generate more realistic images by learning latent sharp images with a similar distribution to clear images. Kupyn et al. [14] proposed a conditional GAN to generate sharper, textured and realistic images. Zhang et al. [15] combined two GAN models, including a learning-to-blur GAN and a learning-to-deblur GAN, to model the natural blurring process in real-world scenarios with sufficient accuracy. However, the generated latent image hardly converges to an ideal sharp image. The network parameters in the test stage are not the ideal Nash equilibrium points. Since the task in the generator is more complex than the discriminator, the discriminator quickly converges to a local optimal solution with few iterations. In contrast, the generator does not converge to the ideal sharp image. It is hard to balance the optimization between generator and discriminator in the training iterations. The distribution gap between training data and the real-world blurred images still exists.

To shrink the distribution gap between training data and real-world images, this paper focuses on balancing the optimization between the generator and the discriminator. To achieve this goal, we try to increase the complexity of the discriminator task to improve the capacity of GAN in image restoration. Image priors are the common properties (such as sparsity) of the latent sharp image and the real-world clear image, which do not depend on specific images to improve the deblurring effectiveness in traditional deblurring methods. Thus, we try to incorporate image priors into the discriminator to increase the complexity. Therefore, based on the generative adversarial network, this paper attempts to incorporate multiple sparse priors into a dual-branch discriminator (DBSGAN) to reduce the dependence on training data. Specifically, this paper proposes a dual-branch architecture in the discriminator based on existing generative adversarial networks. One branch is used to distinguish fake images from real images. The other is used to distinguish the different sparsity between fake and real images. In order to balance the training of the generator and the discriminator, we present a new training strategy for our DBSGAN. The main contributions of this paper are as follows:

- We propose a dual-branch GAN, one branch to distinguish the fake and real image with the other to describe the sparsity of the fake and real image for more realistic image deblurring. In the multiple sparse priors discriminator, we build a sparse constrain model which can have the same optimization with the other branch.
- We design a new training strategy by sharing the network architectures and weights in the dual-branch discriminator to solve the convergence inconsistency between the generator and the discriminator. Furthermore, we alternately iterate the two branches of the discriminator to balance the game between the generator and the discriminator.
- We evaluate our proposed method on both synthetic dataset GOPRO and real-world dataset RealBlur. Extensive experiments demonstrate the superiority of the proposed method over the compared state-of-the-art methods.

The rest of the paper is organized as follows. We first recall the related works about sparse prior-based and deep-learning-based image deblurring in Section 2. We then design our DBSGAN deblurring framework and introduce our dual-branch discriminator with multiple sparse priors in Section 3. Various experimental settings, results and analysis are demonstrated in Section 4. Finally, we discuss and conclude our work in Section 5 and Section 6, respectively.

## 2. Related Work

In this section, we briefly review the significantly related works from the aspect of sparse prior-based and deep-learning-based for image deblurring.

### 2.1. Sparse Prior-Based Image Deblurring

Many methods have been proposed for single image deblurring based on the intrinsic sparsity property of the clear nature images [16,17,18,19,20,21,22]. In order to find a reasonable sparsity of nature images, researchers have explored different vector norms such as the constraint of the latent sharp image in Equation (2). For example, Perrone et al. [21] proposed a total variation (TV) model by $\ell_{1}$-norm of gradient image as the regularization to estimate a latent sharp image. Xu et al. [8] used hyper Laplacian prior ($\ell_{0.5}$) to fit the long tail distribution of a nature image’s gradients and obtain more robust results. Pan et al. [22] adopted $\ell_{0}$-norm on both image and gradient domains for text image deconvolution. Chen et al. [23] used an enhanced sparse model including $\ell_{0}$ and $\ell_{1}$ to regularize image gradients. Zuo et al. [24] used a series of adaptive $\ell_{p}$-norms as the priors and regularizations to improve the performance.

The other sparsity representation of nature’s sharp image has been generalized and modeled to restore the blurry image. Cho and Lee [16] obtained a sharp prediction of the latent image by the gradient and Gaussian priors. In [25], Sun et al. introduced patch priors from nature images which can choose a clear image from blurry ones to facilitate the kernel estimation. The dark channel prior was also absorbed into a non-convex non-linear optimization to deal with natural, face, text and low-illumination images [9]. After that, the extreme priors (including bright channel prior and dark channel prior) [26] which favor clear images over blurry images have also been proposed to obtain a sharp latent image. Recently, Bai et al. [27] introduced a multi-scale latent structure prior under a general down-sampling operation to estimate the latent sharp image.

Most sparse priors represent the statistical features of specific observation images. To reduce the solving difficulty, researchers prefer a simple formulation to describe the complex statistical properties and degradation. Thus, these methods usually perform well on the synthetic blurred images with similar sparsity under simple models. As for the complex real-world image degradation, it is hard to deal with well. Moreover, various priors are sensible for parameters, easily leading to the undesired trivial solution.

### 2.2. Deep-Learning-Based Image Deblurring

Different from designing sparse priors as optimization models, recent various deep-learning-based methods have been proposed by learning a complex imaging model by network architectures and largely collected training data [10,11,12,13,14,15,28,29].

Nah et al. [10] designed the first end-to-end multi-scale network architecture to recover the blurred image with a multi-scale generative adversarial loss function. Therefore, deep networks have become the mainstream method for image deblurring. Gong et al. [30] trained a full convolutional network (FCN) in view of estimated motion flow from the blurred image to obtain the restored result directly. Gao et al. [13] presented a nested ship connection structure for the feature extracted modules and designed the parameter selective sharing mechanism in the whole network, which brings better performance on the Gopro dataset. Zhang et al. [12] proposed a deep hierarchical multi-patch network inspired by Spatial Pyramid Matching to deal with blurry images via a fine-to-coarse hierarchical representation. Last year, Cho et al. [29] re-thought the multi-scale strategy and adopted a new multi-input and multi-output network to improve the quantitative of restored sharp images. All of these methods train their networks based on the discretized synthetic images which are more real than the synthetic images by Equation (1). Thus they perform better than sparse prior-based models. However, these methods do not deal well with real blurred imagesbecause of the gap between the discretized synthetic images and the real-world images.

Recently, some popular network architectures have also been adopted for restoring real-world blurred images. Kupyn et al. [31] proposed DeblurGAN for motion deblurring based on a conditional GAN and the content loss. By adding a double-scale discriminator to a feature pyramid network (FPN) instead of a CNN, Kypyn et al. [14] updated the DeblurGAN to DeblurGAN-V2 version. Wang et al. [32] adopted an effective transformer-based architecture for image deblurring by building a hierarchical encoder–decoder network using the transformer block and obtained a state-of-the-art performance. Transform-based work [33] is difficult to reproduce and improve due to its high requirements on hardware devices such as GPUs. Although the GAN-based method can retain many image textures and look more realistic, the performance of the generator cannot be well played due to the asynchronous convergence between the generator and the discriminator. Therefore, this paper aims to propose an improved GAN to mitigate the training difficulty in image deblurring.

## 3. Our Approach

### 3.1. Our Framework

**Figure 1:**
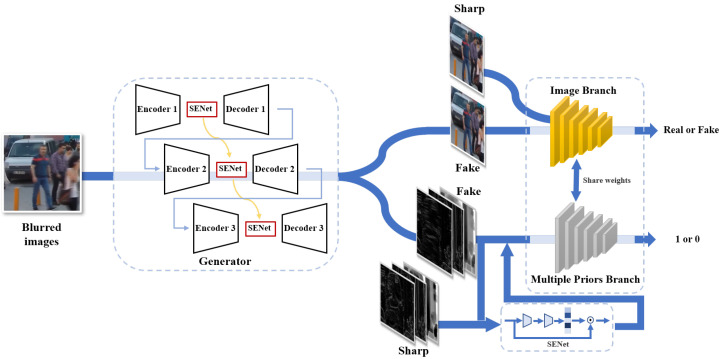
The illustration of our DBSGAN deblurring framework. The generator on the left is a multi-scale encode–decode module. The right part is our dual-branch discriminator which includes an image branch and a multiple-prior branch.

In GAN-based image restoration methods, the generator can learn the mapping from the degraded image to the clear one through the game between the generator and the discriminator. However, due to the different difficulty degrees of the tasks corresponding to the generator and the discriminator, their convergence rate is inconsistent in practical applications. In most cases, the model collapses due to premature convergence of the discriminator, leaving the game in a local Nash solution rather than the sharp picture we desired. The simple idea is that we adjust the game by increasing the training difficulty of the discriminator. It can slow down the convergence of the discriminator and make it jump out of unreasonable local Nash solutions. Therefore, this paper proposes a dual-branch discriminator, which can discriminate image authenticity and distinguish image sparsity simultaneously. Then, we present a dual-branch GAN using various image sparse priors. The network consists of a generative module and consists of two discriminative modules. The architectures are shown in Figure 1 and will be explained in detail as the following sections.

Following [10,29], we consider using a multi-scale encoder–decoder network as a generator to learn the mapping from blurred images to sharp images with rich textures. We formulate the generator as

(3) $$x_{G}=G\left(b;\theta_{G}\right)$$

where G denotes the generator and $\theta_{G}$ is the learnable parameters in the generator. Specifically, as shown on the left of Figure 1 we adopt three-scale encoder–decoder networks to achieve coarse-grained to fine-grained image restoration in stages. The encoder consists of 15 convolutional layers, 6 residual connections and 6 ReLU activation layers. The decoder is symmetric to the encoder except for using deconvolutional layers instead of convolutional layers to generate images. In order to better fuse multi-scale features, this paper adds an attention module (SENet) to the feature layer so that the features of different scales can be fused adaptively to transfer information.

We propose a discriminator which has two branches to alleviate the faster convergence. On the premise of discriminating between the generated image and the real image, we add an auxiliary branch to disturb the optimization of the original discriminator (as shown on the right of Figure 1). The model of the dual-branch discriminator is more complex than the original one. Thus it can slow down the discriminator’s convergent rate. In the auxiliary branch, we take the various knowledge of the image and construct the feature difference between the generated fake image and the real image. Detailed expression is in the following subsection.

### 3.2. Dual-Branch Discriminator with Multiple Sparse Priors

Aiming at the convergence problem of the discriminator in the image restoration task, the purpose of our dual-branch discriminator is to slow down its convergence rate. Then we achieve a game balance between the generative and adversarial modules in the training stage. Therefore, the two branches of the adversarial network need to have relatively similar goals. In other words, they should have close to optimal solutions.

First, we adopt the traditional adversarial between ground-truth sharp images and generated fake sharp images as the first branch of our dual-branch discriminator. It can be described as

(4) $$y_{D_{T}}=D_{T}\left(x_{G};\theta_{D_{T}}\right)$$

where $y_{D_{T}}$ is the prediction of fake or real, and $D_{T}$ and $\theta_{D_{T}}$ are the discrimination network and corresponding learnable parameters, respectively.

The image sparse prior is a vital inherent property in sharp images. Studies have shown that clear images have more sparse gradients, dark channels and other features than blurred images. By integrating these multiple features into the adversarial module as another critical basis of the discriminator, we can achieve image discrimination based on sparse priors. Specifically, we formulate the sparse-based branch as follows,

(5) $$y_{D_{S}}=D_{S}\left(S\left(x_{G}\right);\theta_{D_{S}}\right)$$

where $y_{D_{S}}$ is the prediction of sparsity, and $D_{S}$ and $\theta_{D_{S}}$ denote the discriminatation and corresponding learnable parameters. $S(x_{G})$ is the sparsity function as follows,

(6) $$S\left(x_{G}\right)=A(C(I-|\nabla x_{G}|,I-K\left(x_{G}\right)))$$

where $\nabla x_{G}$ and $K(x_{G})$ denote the gradient and dark channel image of $x_{G}$, I denotes the matrix in which all elements are 1, |·| denotes the absolute value function, and C and A represent the concatenation and attention operators, respectively. The attention operator A implements channel attention by using SENet [34] as the warming-up of gradient and dark channel related features. It is worth noting that we feed $I-|\nabla x_{G}|$ and $I-K(x_{G})$ into the sparse-based branch, rather than the gradient and dark channel image itself. The gradient and dark channel features of clear images are clearer than blurred images, so their corresponding response values will be closer to 0. However, the label of the ground-truth sharp image is one in the first branch. In order to ensure the consistency of the optimal solutions of the two branches, we need to make the label of the groud-truth image in the sparse-based branch also one through the operation in Equation (6).

### 3.3. Training Loss and Strategies

We adopt the relativistic “wrapping” [14,35], which can make the discriminator estimate the probability that the given real data is more realistic than fake data, on the LSGAN cost function [36] to train our dual-branch discriminator. The adversarial loss of the first branch is as follows,

(7) $$\begin{array}{c} L_{D_{T}}=E_{x∼P_{data}\left(x\right)}\left[{\left(D_{T}\left(x\right)-E_{b∼P_{b}\left(b\right)}D_{T}\left(G\left(b\right)\right)-1\right)}^{2}\right]\\ \;+E_{b∼P_{b}\left(b\right)}\left[{\left(D_{T}\left(G\left(b\right)\right)-E_{x∼P_{data}\left(x\right)}D_{T}\left(x\right)+1\right)}^{2}\right] \end{array}$$

where $P_{data}\left(x\right)$ and $P_{b}\left(b\right)$ are the distribution of real-world clear images and synthetic blurred images, respectively. G and $D_{T}$ are the generator and discriminator described in Equations (3) and (4). The adversarial loss of the sparse-based branch is

(8) $$\begin{array}{c} L_{D_{S}}=E_{x∼P_{data}\left(x\right)}\left[{\left(D_{S}\left(S\left(x\right)\right)-E_{b∼P_{b}\left(b\right)}D_{S}\left(S\left(G\left(b\right)\right)\right)-1\right)}^{2}\right]\\ \;+E_{b∼P_{b}\left(b\right)}\left[{\left(D_{S}\left(S\left(G\left(b\right)\right)\right)-E_{x∼P_{data}\left(x\right)}D_{S}\left(x\right)+1\right)}^{2}\right] \end{array}$$

where S and $D_{S}$ are the sparsity function and corresponding discriminator. The adversarial loss makes the training more stable than the WGAN-GP objective [31,35]. We also use Equations (7) and (8) to optimize our generator by learning the parameters of G.

To preserve more realistic color and textures in the generator, as in most approaches, we adopt pixel level and feature level reconstructed errors between the fake and the ground-truth image. We integrate these constraints and use a hybrid loss to train the generator:

(9) $$\begin{array}{c} L_{G}=L_{p}+0.0005*L_{x}+0.001*L_{D_{T}}+0.1*L_{D_{S}} \end{array}$$

where $L_{p}$ is the $\ell_{1}$ norm of the error between the fake and ground-truth image, and $L_{x}$ is the $\ell_{2}$ norm on the VGG19 feature map between the fake and ground-truth one. Based on Equations (7)–(9), how to train our deep network is also crucial.

Traditional generative adversarial networks are usually trained alternately with a discriminator and a generator, and we are no exception. Nevertheless, the difference is that we introduce a dual-branch discriminant network. Suppose we adopt different network architectures of $D_{T}$ and $D_{S}$ and simultaneously optimize them in one iteration. In that case, it means that we reinforce the constraints of the discriminator, which will lead to a faster convergence rate. Therefore, we slow down the convergence of the discriminator from the following two aspects:

- In our dual-branch discriminator, the two branches adopt the same network architectures and share the weights to complicate the discriminator task;
- We alternately optimize the two branches to decrease the discriminator’s convergence rate. Further, we balance the game between the discriminator and the generator.

**Figure 2:**
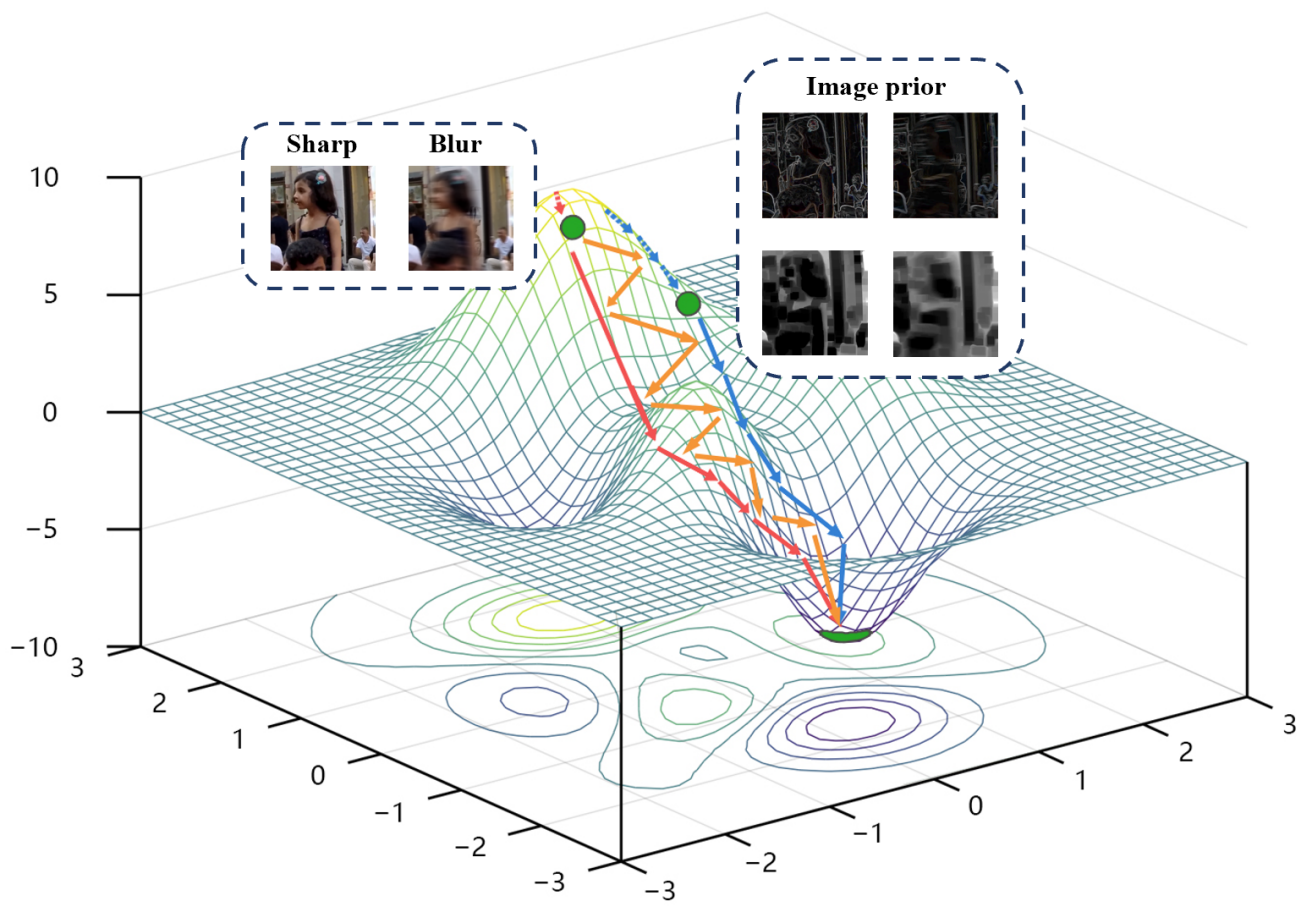
The schematic diagram of convergence curves for different discriminators. The red and blue curves represent only the simple image discriminator or sparse-prior discriminator. The yellow curve is the convergence route of our dual-branch discriminator.

To achieve these requirements, $D_{T}$ and $D_{S}$ will share the network architectures and parameters (denote as $\theta_{D}$). The yellow line in Figure 2 represents the convergence process of our method. By alternately updating the two branch networks with the same optimal point but inconsistent optimization objective functions, the convergence speed can be slowed down on the premise that the optimal solution remains unchanged. The specific training strategy is shown in Algorithm 1. In the training stage, we improve the efficiency with multi-scale networks, the large batch size, a range of learning rates, Adam optimizer and so on. More training strategies are presented in Section 4.1.2. In the testing stage, the trained parameter $\theta_{G}$ from Algorithm 1 is fed into the generator to obtain the deblurred sharp image.
**Algorithm 1:** The training processing of DBSGAN**Require:** Dataset $\left\{x_{i},b_{i}\right\}$, i∈{1,2,⋯,N}, Learnable parameters $\theta_{G}$, $\theta_{D}$.1: **for** k=0,1,2,⋯,K−1 **do**2:    **for** i=1,2,⋯,N **do**3:      $x_{G}\leftarrow G\left(b;\theta_{G}\right)$;4:      $y_{D_{T}}\leftarrow D_{T}\left(x_{G};\theta_{D}\right)$;5:      $\theta_{G}\leftarrow$ Optimize $L_{G}$ by Adam;6:      $\theta_{D}\leftarrow$ Optimize $L_{D_{T}}$ by Adam;7:      $x_{G}\leftarrow G\left(b;\theta_{G}\right)$;8:      $y_{D_{S}}\leftarrow D_{S}\left(S\left(x_{G}\right);\theta_{D}\right)$;9:      $\theta_{G}\leftarrow$ Optimize $L_{G}$ by Adam;10:     $\theta_{D}\leftarrow$ Optimize $L_{D_{S}}$ by Adam;11:    **end for**12: **end for**

## 4. Experimental Evaluation

### 4.1. Experiment Settings

#### 4.1.1. Datasets

We used the GOPRO dataset [10] to train our DBSGAN. The clear images are saved as the video sequence and captured by a GOPRO4 Hero camera with 240 frames per second. The blurry images are generated by averaging successive short exposure frames. Among the 3214 blurry/sharp paired images, we used 2103 pairs for training and the other 1111 pairs for testing.

We also tested on the RealBlur Dataset [37] to verify the effectiveness of our proposed method. It is a real-world blur dataset introduced by Rim et al. The dataset consists of 4738 pairs of images, including the reference images from 232 different scenes. All images are captured in original camera and JPEG format, thus generating two datasets: RealBlur-R for original images and RealBlur-J for JPEG images. Each training set consists of 3758 image pairs, while each test set consists of 980 image pairs.

#### 4.1.2. Implementation Details

We randomly cropped an image with an original pixel size of 1280×720 to 256×256 patches during the training stage. Data augmentation was performed by random horizontal flipping and random $90^{\circ }$ rotation. Then, we reshaped the cropped patches with three scales (1, $\frac{1}{2}\times \frac{1}{2}$, $\frac{1}{4}\times \frac{1}{4}$) and fed them into the generator as the input of each scale. We normalized the image to the range [0,1] and then subtracted 0.5 to make the range in [−0.5,0.5]. The batch size was set to 8, and the Adam optimizer was adopted to train the model for 3000 epochs. The learning rate ranged from $1\times 10^{-4}$ to $1\times 10^{-7}$ with the Cosine Annealing LR strategy. Our experiments were conducted on a workstation with ADM 2950X 16-Core Process CPU and two NVIDIA GeForce RTX 3060 GPU by the Pytorch framework.

### 4.2. Ablation Analysis

**Figure 3:**
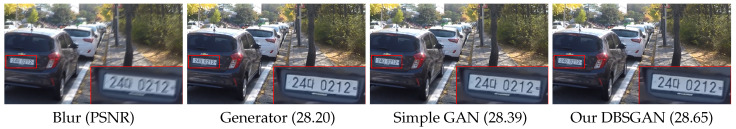
Visual comparisons of different components of DBSGAN on an example blurry image. Quantitative metric PSNR are reported below each result.

Our dual-branch discriminator plays a vital role in our approach. In order to verify its effectiveness in improving image deblurring performance, we analyzed the performances of each component of our DBSGAN. We evaluated the discriminator without itself or each branch of it. For comparison, a baseline model was trained only by our proposed generator, resulting in the averaged PSNR and SSIM on randomly selected images of the GOPRO test dataset. For verification, we repeated the random selection experiment five times and selected 500 images each time. The quantitative results of each time and the average of five times are reported in Table 1. The averaged row includes mean and variance. It can be seen that our DBSGAN is relatively stable with a higher mean and minor variance. We also reported the averaged PSNR and SSIM of all testing samples in the GOPRO dataset, which is listed in the last row in Table 1. It can be seen that our DBSGAN obtains the superior PSNR and SSIM over the simple generator and DBSGAN with one branch. The PSNR of the DBSGAN with only the image branch (i.e., a simple GAN including generator and image branch) improves 0.2 dB compared with the baseline. Similarly, The PSNR of the DBSGAN with only the multiple-prior branch (i.e., a simple GAN including generator and multiple-prior branch) improves 0.1 dB over the baseline. It means that the effectiveness of our multiple-prior branch is limited compared to the image branch. However, the network achieved 0.33 dB higher PSNR than the baseline when incorporating both the image and the multiple-prior branches. The compared qualitative results are shown in Figure 3. Figure 3 provides visible results of each component. It can be seen that our dual-branch discriminator can retain more details around the license plate number.

**Figure 4:**
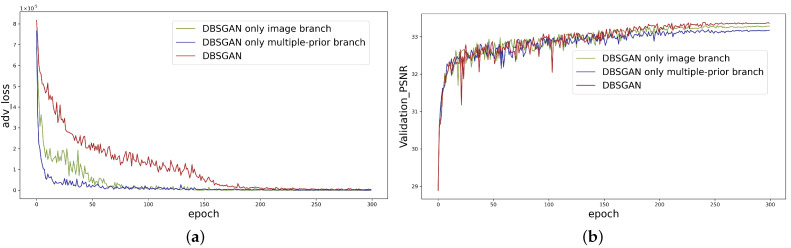
The comparison of adversarial loss between our DBSGAN and the simple GAN with only one branch. (**a**) The convergence performance of adversarial loss in each epoch. (**b**) The PSNR of the validation set in each epoch.

To analyze the performance of the discriminator in our framework, we compared our DBSGAN with the simple GAN with only one branch discriminator. The adversarial loss and PSNR are reported in Figure 4. Our DBSGAN presents higher adversarial loss than the simple GAN with only one branch, which illustrates that the convergence rate is slowing down using our training strategy in Algorithm 1. Furthermore, our method has a similar convergent trend to the other two strategies although our discriminator task is more complex. The PSNR of the validation set (Figure 4b) can also verify that our DBSGAN achieves a higher PSNR than the other two strategies.

### 4.3. Performance on GoPro Dataset

**Figure 5:**
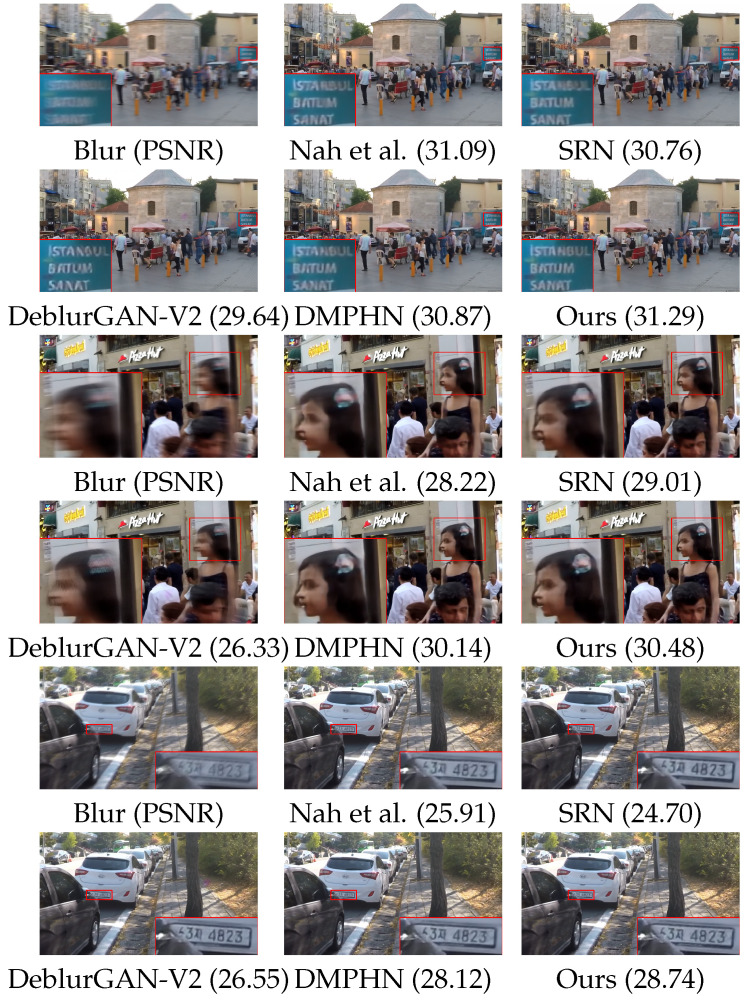
Visual comparisons with state-of-the-art methods on example synthetic blurry images. Quantitative metric PSNR is reported below each result.

We compared our DBSGAN with state-of-the-art image deblurring appro- aches [10,11,12,14,38,39]. We evaluated all the methods with specified or pre-trained model parameters provided by the authors. Table 2 shows the quantitative results of these methods. Our DBSGAN obtained the highest PSNR and SSIM compared with the others. Specifically, our method has a similar generator with DMPHN, while our method improved by 0.5 dB. The qualitative results are presented in Figure 5. It is shown that our model effectively recovers image edges and textures without noticeable blur. In the second group of restored images, the hair accessories on the girl’s head are more precise in our results compared to other methods. Overall, our results are superior to the other competitive methods on the GOPRO dataset.

### 4.4. Performance on RealBlur Dataset

**Figure 6:**
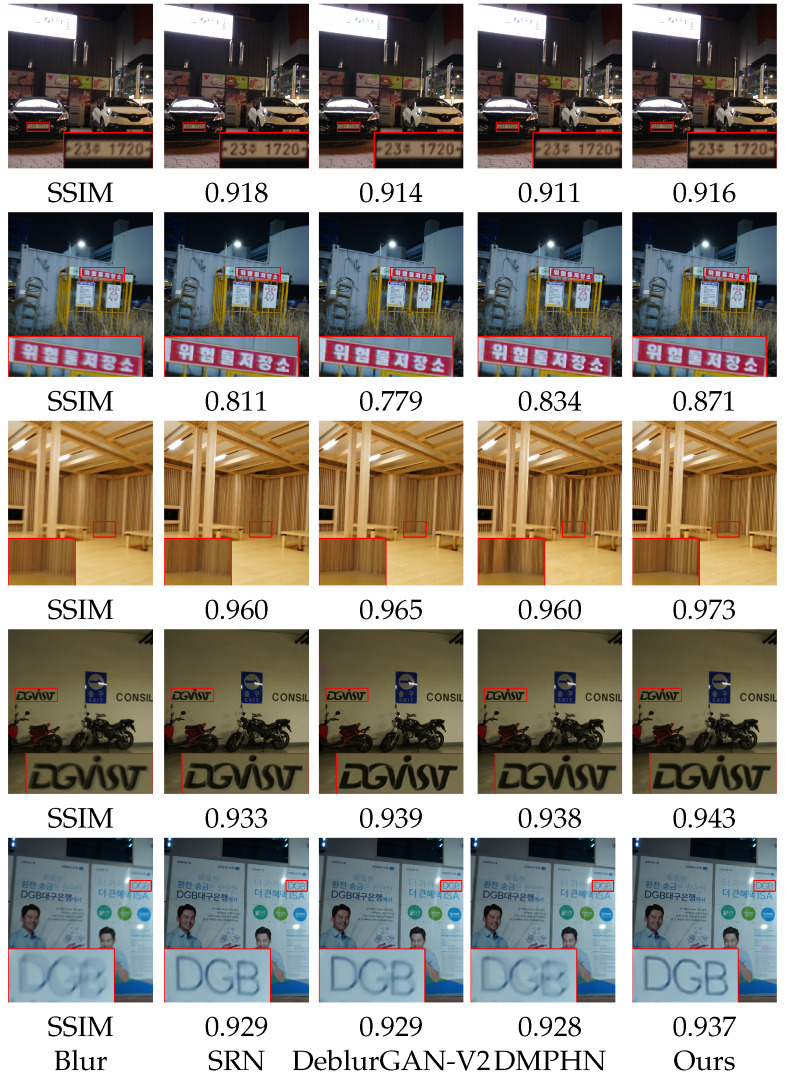
Visual comparisons with state-of-the-art methods on example real-word blurry images. Quantitative metric SSIM is reported below each result.

To verify the performance on real blurry images, we also evaluated our method on the Realblur Dataset [37]. The pre-trained model on the GOPRO dataset was directly applied to RealBlur, and the competitive approaches on the GOPRO dataset [10,11,12,14] are compared. As shown in Table 3, we compared PSNR and SSIM on the Realblur-R dataset to verify the robustness of our DBSGAN. Our method shows the best performance on the most real low-light blurry images in the Realblur-R dataset. Figure 6 shows the visible result both on RealBlur-R and RealBlur-J datasets. It can be seen that our restorations are more sharpened than others regardless of matter light.

## 5. Discussion

It is worth discussing that the experimental effect is not significantly improved. This paper has proposed an improved dual-branch discrimination network and its training algorithm, which can make the training relatively stable. Our ablation analysis can verify it. As the quantitative results are not significantly improved, we believe it is worth further study. On the one hand, because there is no convergence proof of the algorithm, it is not easy to analyze why it happens in theory. In addition, we have investigated the application of a dual-branch discriminator network in other tasks. For example, Ma et al. [40] proposed a threshold for the number of iterations to train the discriminator more times in one epoch. However, how to design the threshold for our proposed dual-branch discriminator network is unclear.

## 6. Conclusions and Future Work

In this paper, we proposed a dual-branch GAN using the multiple sparse priors as auxiliary discrimination of traditional GAN. Instead of only distinguishing the fake and real image in a simple discriminator, we integrate it with a multiple sparse priors branch to build a dual-branch discriminator, enabling us to balance the game between generator and discriminator. Furthermore, we also designed an algorithm that synchronizes the convergence of generator and discriminator by alternating iterations of two branches. The experimental results demonstrate that our method outperforms the other state-of-the-art methods in regard to the quantitative and qualitative results, especially for the GOPRO dataset, the average PSNR improves 1.7% over others.

Although the dual-branch discriminative network proposed in this paper changes the game between the generator and the discriminator in the training stage, the quantitative results of our method are not significantly improved compared to state-of-the-art methods. In terms of training strategy, this paper only provides an optimization method in which two branches alternate. However, how to make network training more effective still needs further exploration. Furthermore, this paper lacks theoretical proof of the algorithm’s convergence, which we should research in future work.

## Figures and Tables

**Table 1:** Performance comparison of different components of DBSGAN.

Generator		✓	✓	✓	✓
Image Branch			✓		✓
Multiple-Prior Branch				✓	✓
1	**PSNR**	30.52	30.70	30.62	30.85
	**SSIM**	0.938	0.939	0.938	0.942
2	**PSNR**	30.43	30.64	30.55	30.77
	**SSIM**	0.936	0.938	0.937	0.940
3	**PSNR**	30.31	30.54	30.45	30.67
	**SSIM**	0.936	0.938	0.937	0.940
4	**PSNR**	30.44	30.63	30.56	30.75
	**SSIM**	0.937	0.939	0.938	0.941
5	**PSNR**	30.71	30.91	30.84	31.04
	**SSIM**	0.941	0.943	0.942	0.945
Averaged	**PSNR**	30.49 ± 0.1466	30.69 ± 0.1421	30.61 ± 0.1454	30.82 ± 0.1401
	**SSIM**	0.938 ± 0.0022	0.939 ± 0.0020	0.938 ± 0.0021	0.942 ± 0.0019
All test data	**PSNR**	30.38	30.58	30.48	30.71
	**SSIM**	0.936	0.938	0.937	0.940

**Table 2:** The average PSNR and SSIM on the GOPRO test dataset.

Method	PSNR	SSIM
**Hyun et al. [38]**	23.64	0.824
**Sun et al. [39]**	24.64	0.843
**Nah et al. [10]**	29.23	0.916
**SRN [11]**	30.24	0.935
**DeblurGAN-V2 [14]**	29.08	0.918
**DMPHN [12]**	30.21	0.934
**Ours**	**30.71**	**0.940**

**Table 3:** Performance comparison on the RealBlur-R dataset.

Method	PSNR	SSIM
**Nah et al. [10]**	32.51	0.841
**SRN [11]**	35.66	0.947
**DeblurGAN-V2 [14]**	35.26	0.944
**DMPHN [12]**	35.70	0.948
**Ours**	**35.83**	**0.952**

## Data Availability

Data underlying the results presented in this paper are not publicly available at this time but may be obtained from the authors upon reasonable request.
